# Trauma and resilience among non-displaced in the early phase of the war in Ukraine

**DOI:** 10.1038/s41598-026-49940-y

**Published:** 2026-04-30

**Authors:** Natalia Bekassow, Stephan Herpertz, Jan Dieris-Hirche, Kostiantyn Polishchuk, Ina Carola Otte

**Affiliations:** 1 Institute for Diversity Medicine, Ruhr University Bochum, Bochum, Germany; 2 Department of Psychosomatic Medicine and Psychotherapy, LWL-University Hospital, Bochum, Germany; 3 Faculty of International Relation, Ivan Franko National University of Lviv, Lviv, Ukraine

**Keywords:** Resilience, PTSD, Armed conflict, Ukraine, Coping, Transnationalism, Health care, Psychology, Psychology

## Abstract

This study investigates how civilians who remained in war-affected suburbs of Kyiv following the 2022 outbreak of war in Ukraine cope with trauma and build resilience under conditions of prolonged insecurity. Using a mixed-methods design, we combine standardized assessments of post-traumatic stress symptoms (PCL-5) with in-depth qualitative interviews conducted in suburbs of Kyiv, a region largely neglected in empirical research. Nineteen interviews were analyzed using qualitative content analysis. Two contrasting case vignettes, one with the highest and one with the lowest PTSD symptom load, were selected to show variations in resilience strategies and resources. Guided by Michael Ungar’s multisystemic social-ecological framework, the analysis explores how resilience unfolds across micro, meso, macro, and transnational dimensions and complements psychological assessments of trauma with a sociological perspective. Rather than viewing resilience as an individual trait, we highlight its relational, structural, and symbolic dimensions. The findings show how civilians sustain meaning and everyday stability under conditions of ongoing conflict, contributing to research on trauma recovery and resilience in active conflict settings.

## Introduction

The outbreak of war in Ukraine, marked by the entry of Russian troops on February 24, 2022, represented a turning point in the region’s recent history and triggered one of the largest displacement movements in Europe since World War II. The suburban towns near Kyiv became emblematic of the early phase of the war and of civilian resilience and were closely monitored by humanitarian agencies and international missions^1–3^.

While over 5.6 million Ukrainians have sought refuge abroad and another 3.7 million have been displaced internally^4,5^, a significant number of civilians have remained in the country, including in areas directly affected during the early stage of the war. Following UNHCR and UNOCHA terminology, “refugees” refer to individuals granted international protection abroad, “asylum-seekers” to those with pending applications, and “internally displaced persons (IDPs)” to those displaced within Ukraine without crossing international borders^6,7^.

Existing literature on trauma and resilience in Ukraine has grown considerably since 2022, including studies on displaced populations^8–11^ and internally displaced persons^11,12^. Far less is known about those who remained in highly affected regions. In this article, we refer to this group as non-displaced civilians, meaning people who remained in their place of residence during the period under study and did not experience internal or cross-border displacement. This study addresses this gap by examining individuals who lived through the early phase of the war in the Kyiv suburbs and chose not to flee.

To our knowledge, no published study has examined the lived experiences of civilians from the Kyiv suburbs during or after the initial phase of the war.

Using a mixed-methods design, this study integrates standardized PTSD screening (PCL-5) with qualitative interviews. Resilience is conceptualised as a multi-level process shaped by individual coping strategies, social support systems, institutional conditions, and transnational ties^13–20^. Two contrasting case studies, representing the highest and lowest PCL-5 scores, are used to explore variation in resilience strategies and resources.

## State of the art

Since the escalation of the war in Ukraine in 2022, research on trauma and resilience has expanded considerably across psychological and sociological disciplines. This includes both a growing number of empirical studies and the adaptation of standardized instruments to the Ukrainian context. For example, Shkolina et al.^21^ validated a Ukrainian version of the CD-RISC-10 resilience scale. More recently, Kurapov et al.^22^ applied a short Ukrainian version of the Brief Resilience Scale (BRS) in their study on the mental health impact of the war six months after the outbreak. Kimhi et al.^23^ further contributed by validating short versions of the Community and Society Resilience Scales (10- and 13-item versions) in Ukrainian.

At the same time, studies consistently report high levels of psychological distress in the Ukrainian population. For example, Chudzicka-Czupała et al.^24^ reported that nearly three-quarters of respondents met diagnostic criteria for PTSD. However, such findings are often based on large but not representative samples, which limits generalizability.

Recent research indicates that mental health outcomes in Ukraine vary significantly depending on individuals’ degree of exposure to extreme and disturbing scenes and their living conditions, echoing findings from other wartime contexts^22^. Numerous studies have since explored PTSD and resilience in displaced populations, including refugees and IDPs^8–11,25^.

By contrast, comparatively little research has examined resilience among populations remaining in Ukraine during the war^26–28^. One such study by Sydorenko et al.^29^ examined resilience among Ukrainian healthcare professionals using the CD-RISC-10 scale. They found high levels of anxiety and depression among respondents (N = 1442), with nearly one-third expressing a need for psychological support. Yet less than 10% had received professional help within the two years following the escalation of the war in Ukraine in 2022. Resilience was negatively correlated with anxiety, depression, and somatic symptoms, with nurses exhibiting lower resilience than physicians.

While the detrimental mental health effects of war and armed conflict have been well documented^30^, comparative evidence suggests that prolonged exposure within active war zones may be as harmful as, or even more harmful than displacement. Wartime studies conducted since the 2000s in Afghanistan, Iraq, and during the Israel–Hamas conflict constantly document high levels of psychological vulnerability among civilians and health professionals exposed to chronic violence and insecurity^31–33^.

## Theoretical framework

### Definition and operationalization of PTSD

The post-traumatic stress disorder (PTSD) is defined according to DSM-5 criteria as a mental condition that may develop following exposure to distressing events involving actual or threatened death, serious injury, or sexual violence, either directly, through witnessing the event, or by learning about such events affecting close others^34^. PTSD symptomatology comprises four clusters: intrusion, avoidance, negative alterations in cognition and mood, and alterations in arousal and reactivity.

### Resilience as a multisystemic and negotiated process

The study builds on Michael Ungar’s multisystemic social-ecological theory of resilience. Drawing on Bronfenbrenner’s^35^ ecological systems theory, resilience is conceptualized as a dynamic interaction between individuals and their social, cultural, and institutional environments^18–20^. It is defined as “both the capacity of individuals to navigate their way to health-sustaining resources, […] and a condition of the individual’s family, community, and culture to provide these health resources and experiences in culturally meaningful ways” [^18^, p. 225].

Following Bronfenbrenner, Ungar^19^ drawing on a systematic synthesis of several decades of resilience research, uses an ecological perspective as a heuristic lens to cluster resilience studies across interrelated systems. These include the microsystem (individuals, families, and peers); mesosystems (relations between these immediate environments); exosystems (institutional and organizational structures) and macrosystems (cultural and political frameworks). These systems are interdependent and non-hierarchical, with adaptive processes emerging dynamically rather than linearly^19,36,37^.

To capture this complexity, Ungar formulates four guiding principles – decentralism, complexity, atypicality, and cultural relativity – which describe how adaptive functioning emerges under diverse social and cultural conditions. These principles informed his later multisystemic understanding of resilience, encompassing interrelated biological, psychological, social, built, and natural systems^38^. While this comprehensive model has been praised for integrating multiple domains of adaptation, scholars have also noted challenges in translating it into empirical research and applied interventions^39^. For this reason, the present study draws primarily on Ungar’s^19^ earlier multisystemic social-ecological framework, which we consider more accessible and analytically productive for qualitative analysis.

### Sociological perspectives on trauma and resilience

To incorporate a sociological perspective, the study draws on Martin Endreß and colleagues. Endreß^40^ argues for a sociology of trauma that treats trauma not merely as a psychological condition but as a social disruption of trust, meaning, and order. He therefore recommends speaking of “traumatizing experience” rather than “traumatic events,” since only consciously remembered experience can be integrated biographically and thus enable sustainable coping [^19^, p. 42]. Until such integration occurs, associated sensations may trigger non-conscious social actions (e.g., avoidance of places) [^40^, p. 47]. Building a phenomenologically grounded sociology of trauma, Endreß outlines five dimensions of disruption: spatial and temporal experience, self- and other-perception, trauma-related memory, and difficulties in articulation. Together, these dimensions describe trauma as a socially embedded rupture. Endreß and Pabst^15^ further emphasize that violence disrupts trust and requires processes of social reconstitution.

In his work on resilience, Endreß conceptualizes it as an interpretive and socially constructed perspective rather than a fixed trait^13^. This perspective focuses on temporality, perceptivity, power, and normative neutrality^16^. Resilience becomes visible ex post, in the reconstruction of reliability and meaning after disruption^41^. These insights guide our analysis and enable to read the cases as processes of social and symbolic re-stabilization, with attention to biography, recognition, and trust.

### Coping and individual stress regulation

At the individual level, the study draws on Lazarus and Folkman’s^42^ transactional model of stress and coping. Coping is defined as a cognitive and behavioral process through which individuals manage internal or external demands appraised as taxing or exceeding their resources. The model distinguishes between problem-focused and emotion-focused strategies and emphasizes the role of appraisal and context.

### Transnationalism as an additional dimension of resilience

To account for global dynamics and cross-border dimensions of resilience, we draw on Ludger Pries’^17,43–47^ concept of transnational social spaces. He defines transnational social spaces as “soziale Verflechtungszusammenhänge”—relational fields that are geographically “diffuse or de-localized” yet stable and that shape social positions and life-worlds beyond national contexts [^43^, p. 456]. These spaces are formed through continuous social practices, such as communication, exchange, and mobility, that link individuals and groups across borders, thereby challenging the idea of the nation-state as the exclusive container of the social.

This perspective builds on the foundational work of N. G. Schiller, L. Basch, and C. S. Blanc^48^, who conceptualize transnationalism as the set of processes by which immigrants build social fields that link together their country of origin and country of settlement. These social fields enable long-distance political, economic, social, and cultural ties, often facilitated by family relations, and provide a sense of continuity and belonging.

In this study, transnational ties are examined as potential resources for psychological stabilization among individuals who remained in war-affected regions.

### Analytical framework

Integrating these perspectives, the study applies a multilevel analytical framework based on Ungar’s multisystemic social-ecological understanding of resilience and Bronfenbrenner’s ecological systems perspective. We use these levels as heuristic categories to explore how adaptive processes unfold across different social and structural contexts. At the *micro level*, the analysis focuses on individual coping strategies and psychological resources. The *meso level* addresses the role of family, peer, and community networks. The *macro level* captures institutional infrastructures, state responses, and public narratives. This framework is extended by a *transnational dimension*, which includes cross-border solidarity, diasporic support systems, and future-oriented mobility options. These levels are analytically distinct but empirically interconnected.

This approach enables a comprehensive understanding of how civilians in war-affected regions of Ukraine mobilize resources, construct meaning, and build resilience under conditions of prolonged insecurity.

**Fig. 1:**
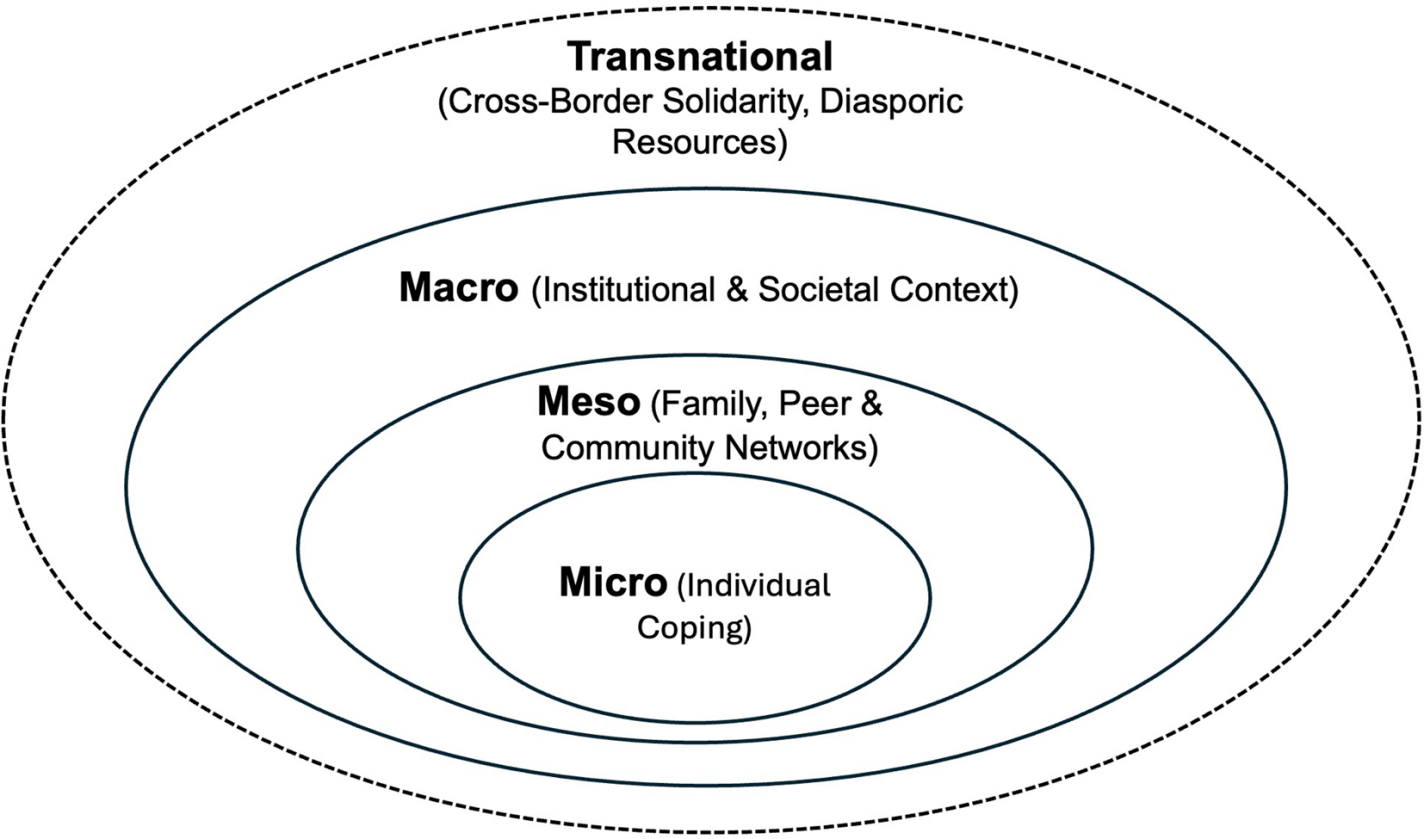
Multilevel analytical framework for examining resilience in war-affected contexts.

Figure 1 provides a schematic overview of this multilevel framework.

## Study objectives and research questions

This study examines how non-displaced civilians in Ukraine experience and process war-related psychological distress and resilience within a multilevel analytical framework.

The research is guided by the following questions:

- How do civilians in Ukraine process their traumatic experiences through coping mechanisms, and how does resilience unfold across micro-, meso-, macro-, and transnational dimensions?
- How do differences in PTSD symptom severity relate to multi-level resilience strategies and resources?

The study provides a context-sensitive perspective on civilian resilience in war settings, extending beyond displacement and pathology-focused approaches. It offers empirical insight into coping and adaptation of civilians in regions, heavily affected during the early phase of the war, highlighting regional differences in exposure.

## Methodology

### Study design

This study follows an explanatory sequential mixed-methods design, in which quantitative data collection precedes the qualitative strand, and the latter has priority in interpretation and analytical depth^49^. The quantitative strand (PCL-5 scores) served primarily descriptive and contextual purposes and guided the selection of two contrasting qualitative cases. This approach aligns with pragmatist perspectives on mixed-methods research^50^.

Integration of quantitative and qualitative strands occurred during data analysis, with qualitative findings interpreted in relation to variations in PCL-5 scores. Symptom severity provided an analytical frame for examining how resilience-related processes were structured and mobilized across cases, without assuming a causal relationship between symptom levels and coping strategies.

### Data collection and sampling

Participants were recruited through an open call disseminated by the local city administration, with logistical support from the office of the Deputy Mayor of Irpin. Participation was fully voluntary, and the administration had no access to participant data. Inclusion criteria comprised adults (≥ 18 years) who resided in Kyiv suburbs of Bucha, Irpin, Hostomel, or Worzel during the early phase of the war and remained non-displaced thereafter. Individuals in acute psychiatric crisis were excluded for ethical reasons.

No formal screening beyond the inclusion and exclusion criteria was conducted. All individuals who met the inclusion criteria and expressed interest participated in the study.

The resulting sample was self-selective, consisting of individuals willing to share their experiences. A total of 19 semi-structured, problem-centered interviews were conducted, following the methodological approach outlined by Flick^51^. Fieldwork took place in July 2024 in the Kyiv suburbs of Bucha, Irpin, Hostomel, and Worzel.

The sample included 13 women and 6 men, partly reflecting mobilization of men during the ongoing conflict. The interviews were conducted by an experienced clinician (SH), with attention to participants’ psychological well-being.

### Quantitative data overview

Quantitative data were collected using the Ukrainian-language validated version of the Post-Traumatic Stress Disorder Checklist for DSM-5 (PCL-5) and a brief sociodemographic questionnaire^52,53^.

The PCL-5 was used as a self-report screening tool, referring to experiences since February 2022 and served to assess symptom severity rather than to establish a clinical diagnosis. For ethical reasons, the participants voluntarily completed the questionnaire after the interview (response rate: 58%). Total scores (range 0–80) were calculated according to the standard procedures^52,53^.

The sample (N = 19) included 13 women and 6 men, aged between 28 and 52 years (M = 40.79; SD = 7.64; median = 41).

Table 1 provides an overview of participants’ characteristics, including gender, age and PCL-5 total scores (where available). Due to ethical considerations and the small sample size, the collection of additional demographic variables was deliberately limited to minimize the risk of re-identification.

**Table 1:** Characteristics of participants.

Participant ID	Gender (M = male, F = female)	Age	PCL-5 score
I1	M	49	45
I2	M	41	70
I3	F	28	31
I4	F	39	62
I5	F	31	36
I6	F	28	n/a
I7	F	32	23
I8	M	51	n/a
I9	F	42	39
I10	M	43	61
I11	F	35	25
I12	F	49	18
I13	F	39	n/a
I14	F	38	37
I15	M	47	n/a
I16	F	52	n/a
I17	F	40	n/a
I18	M	50	n/a
I19	F	41	n/a

Out of 19 participants, 11 provided valid PCL-5 responses. Eight participants did not complete the questionnaire: six declined due to time constraints, and two stated that they were certain they did not suffer from post-traumatic stress and therefore saw no need to fill it in. PCL-5 scores ranged from 18 to 70 (M = 40.6; SD = 17.1; median = 37), indicating substantial heterogeneity. Three respondents (27%) reported high symptom severity (PCL-5 > 50), while 4 people (36%) showed moderate levels (34–50), and four (36%) exhibited low symptom levels (≤ 33). PTSD symptom severity categories were derived using the established PCL-5 cut-off for elevated symptom severity, with additional categories defined for analytical purposes.

**Fig. 2:**
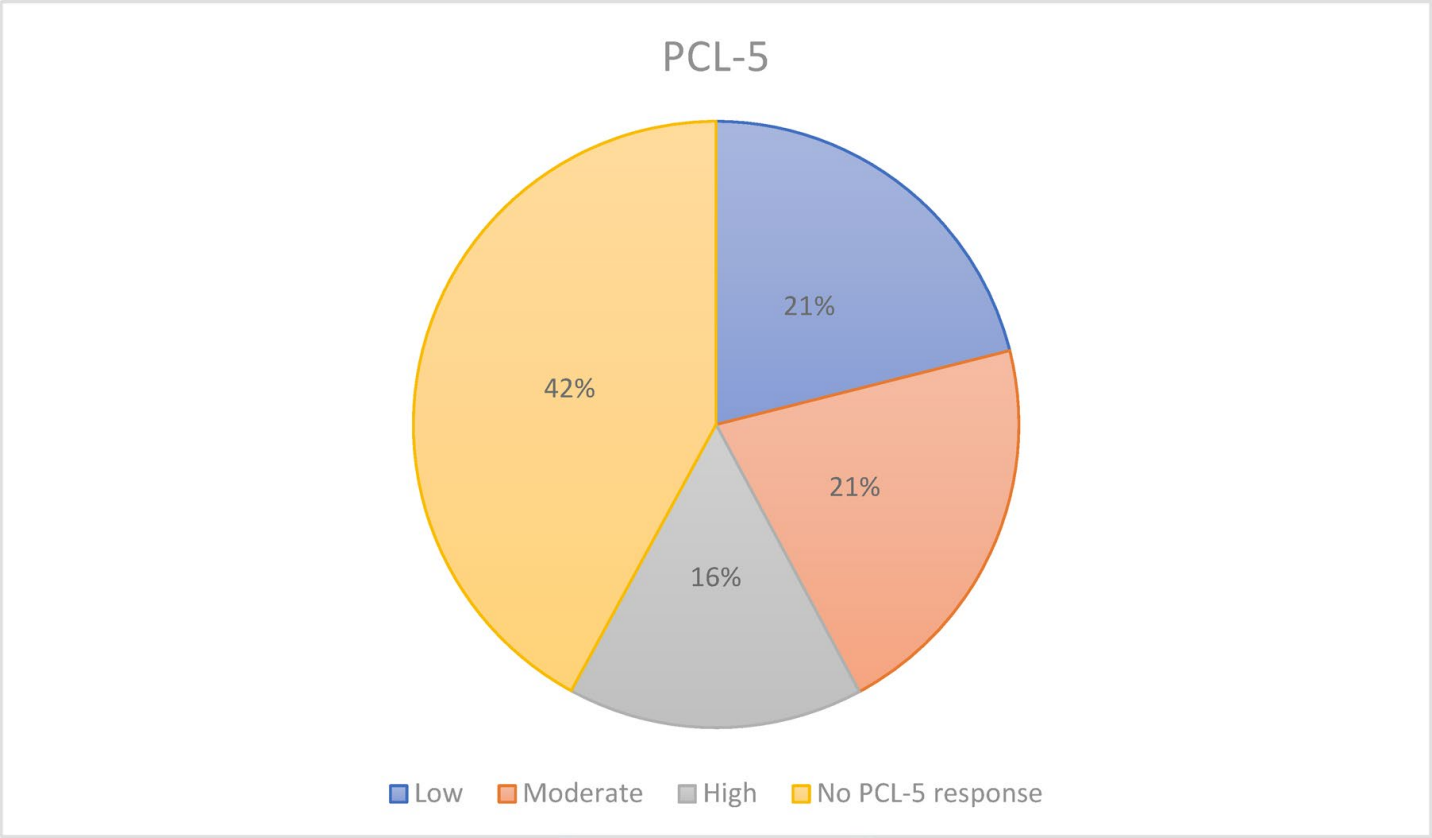
Distribution of PCL-5 responses.

Figure 2 shows the distribution of PCL-5 symptom severity category, including missing responses, in the sample.

These quantitative data were used to contextualize qualitative findings and to guide the contrastive case selection strategy.

Table 2 summarizes the quantitative characteristics of the sample (n = 19).

**Table 2:** Quantitative characteristics of the sample (n = 19).

Variable	n	Mean (SD)/n (%)	Range
Age (years)	19	40.79 (7.64)	28–52
Gender	19		
Female	13	68.4%	–
Male	6	31.6%	–
PCL-5 total score	11	40.6 (17.1)	18–70
PTSD symptom severity category (valid PCL-5 responses)	11		
Low	4	36.4%	–
Moderate	4	36.4%	–
High	3	27.3%	–
Missing PCL-5 responses (out of total sample, n=19)	8	42.1%	–

### Qualitative content analysis

The qualitative data were analysed using a combined inductive-deductive approach, following the principles of Qualitative Content Analysis (QCA) as described by Kuckartz^54^. A thematic-structuring approach was applied to examine resilience processes in crisis settings.

While the interview guide provided an initial deductive frame based on trauma and coping constructs, resilience-related themes were developed inductively from the data. In a first step, open coding was conducted using MAXQDA to identify recurring themes and patterns. These were then structured deductively in relation to the theoretical framework.

The iterative movement between inductive and deductive analysis ensured that theoretical constructs informed but did not predetermine the analysis. To enhance intra-coder reliability, a second round of coding was conducted by the same researcher after a one-month interval^54^.

### Ethical considerations

Ethical approval for this study was obtained from the ethics committee of the Medical Association of Westphalia-Lippe (no. 2024-380-f-N). All participants provided informed consent after receiving a full explanation of the study’s purpose, procedures, and data protection measures. Identifying information was pseudonymized to ensure confidentiality.

Interviews were conducted in line with trauma-informed research principles. Participants were informed that they could pause or discontinue the interview at any point without giving a reason. The interviewer monitored participants’ emotional state throughout, and a Ukrainian-speaking psychotherapist based in Germany was available via teleconsultation in case of acute distress.

The interviewer (SH) is a medical doctor specialising in psychosomatic medicine. Participants were informed that the interviews were conducted solely for research purposes and did not constitute therapeutic sessions. No therapeutic follow-up or interventions were offered. The potential influence of the interviewer’s dual role was addressed through reflexive practice within the interdisciplinary research team.

The semi-structured interview guide covered experiences of trauma, coping, social support, and meaning-making, and can be made available upon request.

## Results

To illustrate central findings from the qualitative analysis, two contrasting case vignettes were selected, based on the evaluation of PCL-5 scores. Using a contrasting sampling approach the case with the highest recorded score (70) and the lowest score (18) were chosen to illustrate differences in distress, coping strategies, and resilience.

These illustrative vignettes exemplify patterns identified across the full sample of 19 interviews.

### Case Vignette: Oleg

#### Background and context

Oleg, a man in his forties and a business owner, has lived with his wife under extreme conditions since the outbreak of the war in 2022. His home was completely burned down during the hostilities.

#### Traumatic experiences and PTSD symptomatology

Oleg shows the highest PCL-5 score (70), well above the clinical threshold for PTSD^52^. He was exposed to continuous psychological stress, particularly through his voluntary involvement in documenting the deceased. This role without institutional or therapeutic support further increases the risk of complex trauma^55^.

He reports symptoms such as chronic exhaustion, apathy, and pessimism about the future:

I suffer more from chronic fatigue.

A large part of the stress comes from not knowing how long it will last.

Taken together, Oleg meets the diagnostic criteria for severe PTSD under the conditions of prolonged exposure.

#### Micro level: individual coping

Oleg’s coping strategy is strongly oriented toward control and action. He describes a structured, deliberate approach:

I tried to do everything deliberately. First a break, then some kind of analysis, then concrete goals. There was no room for emotions, for a nervous breakdown.

This reflects problem-focused coping strategies aimed at restoring control^42^.

From a socio-ecological perspective, this can be understood as an attempt to maintain agency under constrained conditions^18–20^. Oleg’s goal orientation and self-discipline temporarily stabilize his capacity to act in an unpredictable environment. This behaviour exemplifies how individuals may rely on self-regulation as a micro-level adaptation when external systems are weakened or absent.

According to Endreß^13,40^, what is considered ‘successful coping’ depends on cultural and normative contexts. Oleg’s effort to maintain discipline and suppress emotions can thus be understood as an attempt to preserve biographical coherence and social normality under conditions of disruption. Traumatic experiences, however, involve a fundamental loss of control and a “shattering” of temporal and spatial continuity^40^. Coping therefore includes not only psychological regulation but also the re-integration of disrupted experiences into biographical and social memory.

While resilience may appear as strength, it can also mask a form of over-adaptation to extreme circumstances that is neither psychologically nor socially sustainable. Oleg’s case illustrates this ambivalence: coping may be effective in short term but limited in sustaining long-term recovery, a phenomenon frequently observed in war and disaster sociology^56^.

#### Meso level: social relationships and collective action

Oleg’s narrative highlights the importance of social relationships in coping with crisis experiences. In particular, his partnership with his wife emerges as a stabilizing resource, which he repeatedly acknowledges during the interview:

The first was probably the support of my wife. […] We supported each other as best we could.

We were always close, side by side, everywhere, together, the two of us.

Social embeddedness is considered a central protective and developmental factor in nearly all resilience research. Following Ungar’s multisystemic social-ecological perspective^18,19^, stable interpersonal relationships represent core micro- and meso-level processes that enable navigation to and negotiation for resources. Such ties are not only sources of emotional support, but also as spaces for agency, meaning-making, and identity stabilization.

Beyond the family, Oleg highlights help from previously unknown individuals:

I have 100 contacts in my phone of people I have never met in my life who gave me so much.

These forms of spontaneous, non-institutionalized assistance reflect a civic space of resilience based on solidarity and shared experience.

#### Macro level: state failure and systemic frustration

Oleg expresses strong disillusionment with state institutions, particularly regarding corruption:

Corruption is very strong and very painful […]. If they steal from the army, that’s absolutely unacceptable.

He condemns corruption especially in a military context, where trust, loyalty, and justice are central to collective survival. Oleg describes a situation in which individuals and civil society actors are forced to compensate for perceived state non-responsibility, leading to long-term exhaustion.

Such macrostructural weaknesses illustrate how resilience depends on reciprocal interaction between individuals and their institutional environments^18,19^. When reliable and accessible systems of protection and support fail, actors experience a double loss: of external order and interpretative coherence^13^. Adaptive capacity must be compensated for on other levels, resulting in cumulative overload under prolonged stress.

In Oleg’s case, the attempt to compensate for institutional dysfunction through civic engagement reflects a socially produced form of overextension rather than empowerment. Prolonged exposure to uncontrollable situations leads to a “rigorous loss of control”, which can only be integrated if collective frameworks of meaning and trust are re-established. His exhaustion thus reflects not a lack of willpower but the absence of structural and symbolic contexts in which individual action could be stabilized^15,40^.

#### Transnational level: hope and cross-border connections

Oleg’s narrative illustrates that resilience can also be sustained through transnational social ties. Although he remains in Ukraine, he refers to potential support from abroad:

If psychological rehabilitation is offered with temporary relocation to Germany (I have a friend in the U.S. who does something like that) […] I’d be more than happy to participate if I can be of help.

The idea of receiving recognition, aid, or safety through international networks functions here as a symbolic resource. Even though Oleg does not intend to flee, the reference to external, international actors provides psychological relief and a sense of having access to a “safe haven”. In line with the concept of transnational social spaces^17,44,45,48,57^, such connections represent “social interweaving relations” that link individuals and communities across national borders through continuous practices of communication, exchange and solidarity. These networks can foster resilience by combining psychological relief (through the imagination of safety), social connection (to global communities), and potential financial support. They demonstrate that resilience articulates dynamically across relational fields that extend beyond national borders, even when those borders are not physically crossed.

### Case Vignette: Irina

#### Background and context

Irina, a woman in her fifties, lives with her husband in Bucha. She works in property management and has adult children and grandchildren.

#### Traumatic experiences and PTSD symptomatology

Although Irina reports direct and indirect experiences of violence, her PCL-5 score of 18 is well below the clinical threshold for a PTSD diagnosis and the lowest in the sample^52^. She describes the occupation as a radical rupture from her previous life:

We collected firewood [...] we cooked when there was no shelling

She also recounts intrusive memories linked to specific places:

In the early days, I couldn’t even walk. I couldn’t go to the park. I saw people’s faces.

Retrospectively, Irina reports symptoms across PTSD clusters, including avoidance, emotional numbing and somatic stress reactions. At the time of the interview, Irina describes psychological stabilization:

I now sleep so soundly that I don’t even hear the air raid siren.

#### Micro level: individual coping and emotional processing

Irina’s coping combines emotional suppression, symbolic meaning-making, and hope. Her narratives revolve around the challenge of maintaining control amidst chaos and fear.

She no longer walks her dog in certain parts of the park, because she keeps seeing the faces of fallen soldiers there:

When you walk past them [the fallen soldiers], you still can’t believe it. Maybe that’s my internal defence mechanism. I don’t let the grief in.

This avoidance exemplifies what Endreß and Pabst^15^ call a “shattered experience of space.” After trauma, previously meaningful environments can become saturated with intrusive memories, leading to avoidance of specific locations. As long as the traumatizing experience has not been integrated into biographical memory, these spatial reactions reflect a temporary loss of control rather than deliberate choice^15^. Irina’s emotional control is largely maintained, but breaks in specific situations, for example, during the funeral of a soldier she knew:

I went once, I couldn’t resist, I trembled, I cried a lot. When I walk past a photo, it’s okay, but when I see a man I personally knew, it’s very hard.

At the same time, Irina stabilizes herself through everyday routines, such as walking her dog:

I work very hard. I have a dog to reduce my stress. When you go for a walk with him, it distracts you.

This corresponds to problem-focused coping as an active attempt to manage stress-inducing situations through structure and action^42^.

A clear future orientation also remains central to her narrative:

I’m waiting for a victory, and I want to go on living and evolving with my husband. I need some kind of future.

Forward-looking perspectives function as a key resource under conditions of prolonged uncertainty^18,19^.

#### Meso level: social relations and intergenerational dynamics

Irina’s narrative also highlights the central role of close relationships and intergenerational dynamics, as a source of stability. Irina describes how she and her husband developed a shared stance toward survival:

My husband and I agreed not to give up. We decided to make the best of it.

Even in situations of physical separation, communication remains a key resource:

When I go for a walk [with my dog], I talk to my husband if I can reach him at the front, or when he calls me. It’s a positive family thing. I show him videos or something else.

At the same time, family relations intensify under conditions of crisis. Although her children are already adults, Irina notes:

On the contrary, we have grown closer here.

These examples illustrate how resilience at the meso level is sustained through emotional closeness, shared orientation, and the maintenance of social bonds across distance.

#### Macro level: national identity, social tensions, and recognition deficits

Irina links her personal experiences to broader societal transformations during the war, including strengthened national belonging and tensions around the recognition of collective suffering. In her narrative, the war appears as a collective rupture, which produces new forms of self-affirmation as well as fragmentation:

For some people, this war may have been an opportunity to go abroad and give their children a better education […] For some, it is grief because they lost someone. For others, it may have given them courage.

For me, the value of my homeland, Ukraine, is what matters most. This large-scale invasion was a step toward realizing that.

These statements reflect a crisis-induced rearticulation of collective identity. Although Irina identifies ethnically as Russian, she experiences the war within a Ukrainian national framework. In Irina’s case, the collective narrative of resistance, national renewal, and rebuilding provides her with a sense of emotional coherence and moral direction. As Endreß and Pabst^15^ argue, traumatic events can shatter one’s experience of self and others; aligning with a new collective identity becomes a way to restore coherence and rebuild trust in the social world.

At the same time, Irina describes emerging social tensions following the return of displaced persons, fuelled by perceived unequal recognition of suffering:

They think we lived here like a gift from heaven […] People don’t understand that we also experienced things we don’t want to remember.

This tension between remembering and deliberate forgetting illustrates what Endreß and Pabst^15^ describe as the fragmented nature of trauma memory. Traumatizing experiences often resist coherent integration into biographical memory and persist as fragmented traces. Irina’s words express this ambivalence, acknowledging what cannot be remembered without reliving it, thus revealing both avoidance and the persistence of unassimilated experience.

Her sense of being unheard exemplifies what Endreß and Pabst^15^ call the social dimension of trauma-related speechlessness. The inability to communicate traumatic experiences reflects a lack of social resonance and recognition rather than an individual failure. In Irina’s account, this lack of mutual understanding between those who stayed and those who fled transforms unspoken suffering into social division.

This dynamic can be further understood in light of Endreß’s^40^ view of trauma as a disruption of social trust that requires communicative acknowledgement. When suffering remains unrecognized, individuals experience renewed exclusion, impeding the reconstruction of social order and shared meaning.

These statements also illustrate that resilience at the macro level depends not only on state support but also on social recognition and validation of lived experiences^18,19^.

### Transnational level: flight, networks, and moral judgments

Irina reflects on the possibility of leaving the country, enabled by transnational ties, as a psychological resource. Although neither she nor her family left Ukraine, this option contributes to her sense of security:

Of course, I told my daughter: If something happens, take my granddaughter and go abroad. We’ll somehow manage here.

The symbolic availability of cross-border safe spaces functions as a mental safety net, even without actual mobility.

Pries^17^ argues that migration and mobility should not be understood merely as physical movement but as dynamic, multifaceted processes in which relationships, meanings, and resources are negotiated across national borders. Even without physical mobility, the transnational ties and diasporic support provide a sense of potential security and agency, thus enhancing the person’s resilience.

### Cross-case findings across analytical levels

#### Micro level

Across the sample, participants employed a wide range of coping strategies in response to prolonged war-related stress, including both problem-focused coping (e.g., structured action, volunteering, rebuilding) and emotion-focused coping (e.g., faith, meaning-making, suppression)^42^.

A recurring theme across interviews was the capacity to integrate traumatic experience into biographical continuity. Several participants described a profound disruption of temporal experience, reporting that time had “stood still” on the day of their most distressing event:

I remained in that day, at that point. I am not moving forward.

Others, however, articulated a future-oriented perspective that counteracted this sense of a “frozen present”^40^. Hope for personal, familial, and collective recovery emerged as an important psychological resource:

I see that our Irpin, our State, is becoming stronger every day. If we maintain our current unity, we will definitely survive.

Religious faith constituted a further relevant coping resource. Several participants described a turn towards faith under conditions of existential threat, particularly within the framework of Ukrainian Orthodoxy:

In the trenches, when you talk to people, there are almost no atheists left. Maybe they aren’t theologians who can explain their faith, but when your life is in danger… then faith appears.

In addition to religious practices, participants highlighted the importance of small, symbolic everyday routines (e.g., putting on lipstick or drinking familiar coffee) helped maintain sense of normalcy and continuity.

#### Meso level

Social relationships were the most consistently reported protective factor. Family members, neighbours, and local networks provided primary sources of emotional support, practical assistance, and a sense of belonging. In almost all narratives, these relationships compensated for absent or insufficient institutional support and formed the central foundation of resilience.

Participants repeatedly described how everyday social relations were transformed during the war, shifting from individualized routines toward collective openness and mutual responsibility, thus promoting resilience. The reconfiguration of social life was also expressed as a deepening of neighbourhood relations:

We know all our neighbours now – not like before, when everyone lived in their own shell, home–work–work–home. Now it’s different: we know our neighbours, we notice new faces in the courtyard, we talk about who moved in and from which apartment. It’s like a big family.

Beyond family and neighbourhood ties, professional and collegial contexts were likewise described as important sources of emotional support and mutual recognition:

Exactly here, among colleagues who work with you, who know your work – and I know what my colleagues are doing – we can give each other this support.

Finally, some participants interpreted the witnessed solidarity as a culturally embedded capacity:

Ukrainians are indeed people who know how to unite and come together, and somehow they manage to achieve something.

Differences in PTSD symptom severity across the sample appear linked to variations in available social and institutional support, underscoring resilience as a multisystemic and context-dependent process.

#### Macro level

Participants frequently reported a perceived absence of government support, particularly during the early phase of reconstruction. Several narratives emphasized that rebuilding efforts relied primarily on civil society initiatives and international assistance:

We rebuilt our center with the help of international partners. Not a single cent came from the government.

While grassroots initiatives and civil society efforts clearly filled some of the resulting gaps, these accounts also highlighted the structural limits of “resilience from below”. In the absence of reliable institutional infrastructures, individuals and communities were often left to shoulder the burden of adaptation on their own.

#### Transnational level

The transnational dimension also emerged as a significant theme in participants’ narratives. Expressions of gratitude for international assistance, either material, moral, or symbolic, suggest that cross-border solidarity offered not only practical resources but also hope and affirmation:

Of course, we also want to thank our international partners. Without them, we wouldn’t have recovered.

Diasporic and international ties thus functioned as resilience-enabling environments that complemented, and in some cases partially substituted for failing domestic systems. Even without physical mobility, the awareness of international solidarity appeared to foster a sense of belonging, visibility, and future orientation among civilians who remained in war-affected regions.

## Discussion

This study examined (1) how non-displaced civilians in Ukraine process traumatic experiences and how resilience unfolds across micro-, meso-, macro-, and transnational dimensions, and (2) the relationship between PTSD symptomatology and multi-level resilience strategies and resources.

Participants mobilized individual, relational, and collective resources, while differences in PTSD symptom severity were closely linked to variations in available social and institutional support. The contrast between Irina and Oleg illustrates how similar exposure can be associated with markedly different psychological outcomes. This supports understanding resilience not as the absence of distress, but as a context-dependent process shaped by available social and structural resources.

From a sociological perspective, these patterns resonate with what Endreß and Pabst^15^ and Endreß^40^ describe as the social dimensions of trauma. Traumatic experiences silence when communication frameworks are absent^40^. In our study, participants found alternative ways to speak about their experiences outside of professional therapy. Many described meetings with neighbours and repeatedly recounting the same memories – stories that “everyone already knew by heart”. This collective retelling can be interpreted not as stagnation, but as a social form of catharsis and meaning-making. In the absence of professional psychological support, such informal conversations created a communal space of trust and recognition, allowing participants to process their experiences together as a form of collective healing. Through repetition and mutual acknowledgement, individual suffering was transformed into shared experience^15^.

Our qualitative findings are consistent with broader empirical research on trauma exposure during the war in Ukraine. The study of Karatzias et al.^27^, for instance, reports elevated rates of PTSD and complex PTSD (cPTSD), with higher exposure associated with increased risk. However, this evidence relies on self-report data collected online and is not based on representative sample, which limits generalizability. Obtaining valid epidemiological data on the prevalence of PTSD and cPTSD with acceptable representativeness remains methodologically challenging, particularly under conditions of ongoing war, where representative sampling is difficult to achieve.

A cross-sectional study in 11 neighbouring countries reported mean PTSD and cPTSD rates of 9.9% and 10.1%, respectively^58^, with risk factors including pre-existing mental disorders, higher anxiety, female gender, stronger impact of war news, lower resilience, and proximity to Ukraine. These findings provide contextual evidence that trauma-related disorders can arise from both direct and indirect experiences, including cumulative “microtraumas”, which may contribute to cPTSD.

At the meso-level, our qualitative findings underscore the central role of family, peer groups, and local solidarity networks as pillars of resilience. This aligns with previous research linking social support to improved mental and physical health outcomes^59,60^ and affirms Monson et al.’s^61^ conclusion that intimate partner support significantly mitigates PTSD symptoms. At the community level, the role of social capital in recovery processes has been emphasized by Abramson et al.^62^, while Endreß^13^ calls for a relational, non-individualized understanding of resilience grounded in practices of care and responsibility.

Extending beyond local and national contexts, participants’ narratives highlight the importance of transnational resilience resources. Recent scholarship shows that armed conflict often activates transnational diasporas, which mobilize around collective memory, solidarity, and moral responsibility^63–65^. In the case of Ukraine, such mobilization has been observed across Europe—e.g. in Sweden^66^, France and Poland^67^,—as older and newer migration waves unite to form cohesive transnational networks of support and advocacy.

For non-displaced civilians, the awareness of diasporic solidarity and the availability of refuge can serve as psychological resources. As Müller-Suleymanova^64^ argues, diasporic memory and moral engagement can transform collective suffering into agency, which, in our interpretation, strengthens the transnational dimension of resilience.

The findings also point to practical implications. Strengthening trauma-informed community support and expanding access to psychosocial interventions, including group-based formats is essential. In contexts of ongoing war, international cooperation in mental health provision should be reinforced.

Finally, the researcher’s roles shaped the study. Interviews were conducted by a researcher with extensive clinical expertise, ensuring sensitivity and trust. The combination of clinical, linguistic and social-scientific perspectives facilitated field access and supported a careful approach to data collection and interpretation, enhancing analytical depth.

## Limitations

This study has several limitations. The self-selected sample may introduce selection bias, as participation required both emotional readiness and logistical accessibility. Participants may therefore differ systematically from those who declined, particularly in their willingness to disclose sensitive experiences. While the findings provide rich context-specific insights, they are not be generalizable to all war-affected populations.

The gender distribution of the sample (13 women and 6 men) is unbalanced. This may be partly explained by the mobilization of men for military service, as well as gendered patterns of disclosure, with women more likely to narrate traumatic experiences as a means of processing^68,69^. These dynamics also vary across cultural settings. This imbalance limits transferability and highlights the need for more gender-balanced research.

In addition, the interviewer’s dual role both as a clinical expert and researcher may have influenced responses. Future studies should consider a multi-interviewer approach to strengthen reflexivity and triangulation.

Despite these limitations, the findings provide insight into the multi-layered nature of resilience among civilians in war-affected context. Using a multisystemic resilience framework, the study shows how individual, social, structural, and transnational resources interact in shaping the lived experiences of non-displaced civilians under conditions of severe crisis.

## Conclusions

This study highlights the value of a multi-level, culturally informed approach to resilience in contexts of prolonged conflict. Drawing on interdisciplinary theory and a mixed-methods design, we show that individual resilience is shaped not only by psychological coping strategies, but also by social relationships, institutional structures, and transnational ties. Rather than reducing resilience to a personal trait, we conceptualize it as a dynamic, negotiated process embedded in lived experience and access to resources.

Our findings support the need for research designs that combine standardized assessment tools with qualitative approaches to better capture how resilience develops under crisis conditions. We argue for geographically specific studies that reflect the uneven impact of war across regions and highlight the value of incorporating a transnational perspective into future resilience research.

## Data Availability

Due to the sensitive and potentially identifying nature of the qualitative data collected in an ongoing conflict zone, the full interview transcripts cannot be shared publicly. The study participants did not provide consent for open data sharing. Anonymized excerpts used in this manuscript are available in the main text and supplementary materials. Further information may be made available from the corresponding author upon reasonable request and subject to ethical review.
